# β-catenin promotes MTX resistance of leukemia cells by down-regulating FPGS expression via NF-κB

**DOI:** 10.1186/s12935-020-01364-y

**Published:** 2020-06-24

**Authors:** Shu-Guang Liu, Zhi-Xia Yue, Zhi-Gang Li, Rui-Dong Zhang, Hu-Yong Zheng, Xiao-Xi Zhao, Chao Gao

**Affiliations:** Beijing Key Laboratory of Pediatric Hematology Oncology, National Key Discipline of Pediatrics, Ministry of Education, Key Laboratory of Major Diseases in Children, Ministry of Education, Hematology Oncology Center, Beijing Children’s Hospital, Capital Medical University, 56 Nanlishi Road, Beijing, 100045 China

**Keywords:** Acute lymphoblastic leukemia, Drug resistance, Folylpolyglutamate synthetase, Methotrexate, β-catenin

## Abstract

**Background:**

Aberrant activation of β-catenin has been shown to play important roles in the chemoresistance of acute lymphoblastic leukemia (ALL), but the involvement and mechanism of β-catenin in methotrexate (MTX) resistance is poorly understood. In the present study, we demonstrate a critical role of β-catenin-NF-κB-FPGS pathway in MTX resistance in the human T-lineage ALL cell lines.

**Methods:**

Lentivirus sh-β-catenin was used to silence the expression of β-catenin. Flow cytometry was performed to detect apoptosis after MTX treatment. Western blot, real-time PCR, Co-immunoprecipitation (Co-IP), Chromatin immunoprecipitation (ChIP), Re-ChIP, and Luciferase assay were utilized to investigate the relationship among β-catenin, nuclear factor (NF)-κB, and folypoly-γ-glutamate synthetase (FPGS).

**Results:**

Depletion of β-catenin significantly increased the cytotoxicity of MTX. At the molecular level, knockdown of β-catenin caused the increase of the protein level of FPGS and NF-κB p65. Furthermore, β-catenin complexed with NF-κB p65 and directly bound to the FPGS promoter to regulate its expression. In addition, β-catenin repression prolonged the protein turnover of FPGS.

**Conclusions:**

Taken together, our results demonstrate that β-catenin may contribute to MTX resistance in leukemia cells via the β-catenin-NF-κB-FPGS pathway, posing β-catenin as a potential target for combination treatments during ALL therapy.

## Introduction

T cell acute lymphoblastic leukemia (T-ALL) is an aggressive hematological malignancy that accounts for about 15% of pediatric acute lymphoblastic leukemia. Despite of significant improvements in therapy, a number of childhood T-ALL patients experience relapse and face a dismal prognosis. Drug resistance is a key factor contributing to relapse [1, 2]. Thus, understanding the molecular basis for chemoresistance may pave the way for improving drug sensitivity and prognosis.

Methotrexate (MTX) is a major component in all contemporary treatment protocols for childhood ALL, but emergence of resistance to MTX remains an obstacle towards curative chemotherapy [3, 4]. Folypoly-γ-glutamate synthetase (FPGS) is responsible for the activation of MTX to MTX polyglutamates (MTXPGs). The ability of leukemia cells to accumulated MTXPGs was shown to be a determinant of MTX activity in childhood ALL patients. Consequently, loss of FPGS activity is a well-established mechanism of resistance to MTX in vitro and in vivo [5–7]. FPGS activity is significantly correlated with *FPGS* mRNA levels. In childhood ALL, a strong correlation exists between *FPGS* expression, intracellular MTXPGs accumulation and treatment outcome. T-ALL patients fare worse than other patients treated with antimetabolite-based chemotherapy regimens, resulting from significantly lower FPGS expression, FPGS activity, and MTXPGs accumulation [8, 9]. There are few previous studies on the FPGS transcription regulation. It is reported that HDAC1 is recruited by NFY-B and Sp1 proteins to the FPGS promoter in a multiprotein complex that includes CBP, to regulate FPGS mRNA expression epigenetically through chromatin remodeling [10]. Some fusion proteins, such as TEL-AML1 and E2A-PBX1, can also suppress FPGS transcription by recruiting co-repressors (mSin3A, Rb) and HDAC1 to the FPGS promoter region [11]. Further study is required to better understanding the mechanisms that control FPGS expression in T-ALL, which could lead to the development of novel targets capable of upregulating FPGS and increasing MTXPGs accumulation.

In order to find novel transcription factors for FPGS regulation, we analyzed the promoter region of FPGS and found two potential NF-κB p65 binding sites, indicating that NF-κB might be a transcription factor for FPGS. Previous study suggested that β-catenin can complex with NF-κB, inhibit NF-κB activity and repress its target genes in human colon and breast cancer cells [12]. β-catenin can also complex with HDAC1 to regulate target genes [13]. So β-catenin is probably involved in the FPGS regulation. It is reported that β-catenin contributes to MTX resistance in osteosarcoma and colorectal cancer. Ma et al. [14] found that β-catenin knockdown increased the sensitivity of osteosarcoma cell line Saos2 to MTX-induced cell death. The Wnt/β-catenin signaling was activated in a MTX-resistant colorectal cell line HT-29 (HT-29-R) [15]. But the role of β-catenin in MTX resistance in ALL has not been clarified.

In this study, we found that β-catenin promoted MTX resistance in T-ALL cells. Mechanistically, we showed that β-catenin can interact with NF-κB p65 and repressed the protein expression of p65. Importantly, β-catenin was found to inhibit the expression of NF-κB p65 target gene FPGS. Thus, our data reveal a novel β-catenin-NF-κB-FPGS pathway involved in MTX resistance and suggest that targeting β-catenin may be a therapeutic strategy for combined therapy.

## Materials and methods

### Cell culture and treatments

The human T-lineage ALL cell line Jurkat, CCRF-CEM, MOLT4, and human embryonic kidney (HEK)-293 were purchased from the cell bank at Peking Union Medical University. Jurkat, MOLT4, and CCRF-CEM were cultured in RPMI 1640 (Invitrogen) supplemented with 10% fetal bovine serum (FBS, Invitrogen), penicillin (100 U/mL), and streptomycin (100 mg/mL). HEK-293 was cultured in Dulbecco’s Modification of Eagle’s Medium with 10% FBS, penicillin (100 U/mL), and streptomycin (100 mg/mL).

hCTNNB1 knockdown was carried out using lentivirus containing shRNA-1 oligonucleotide: 5′-TTGGAATGAGACTGCTGAT-3′ and shRNA-2 oligonucleotide: 5′-CATGGAAGAAATAGTTGAA-3′. A scrambled shRNA lentivirus with no effect on hCTNNB1 was constructed as a negative control using the following sequence: TTCTCCGAACGTGTCACGT (GeneChem Co. Ltd. China).

Jurkat was treated with the NF-κB inhibitor, pyrrolidine dithiocarbamate (PDTC; 100 μM; Sigma-Aldrich), NF-κB stimulant lipopolysaccharides (LPS; 1 μg/mL; Sigma-Aldrich), and Canonical WNT signaling inhibitor PRI-724 (20 μM; Selleck) respectively for 20 h to investigate whether FPGS was regulated by the NF-κB and WNT pathway. To examine the effect of β-catenin on MTX resistance, cells were treated for 24 h with 10 μM MTX at 48 h posttransfection of RNAi lentivirus. To examine the protein turnover of FPGS, cells were treated with a protein biosynthesis inhibitor cycloheximide (CHX, MCE) at a final concentration of 50 μg/mL for 0, 2, 4, and 8 h, respectively.

### Sample preparation

Bone marrow (BM) samples of patients were collected at diagnosis. Mononuclear cell separation was carried out as previously described [16] and immediately stored at − 70 °C until use. This study was approved by the BCH Institutional Ethics Committee and informed consent according to the Declaration of Helsinki was signed by the guardians of the patients.

### RNA isolation and real-time PCR

Total RNA was isolated using Trizol reagent (Invitrogen) according to the manufacturer’s instructions. Isolated RNA was used as a template for the reverse transcription reaction (Invitrogen). Quantitative real-time PCR analysis was carried out using PowerUP SYBR Green Master Mix (Applied Biosystems) on ViiA 7 System (Applied Biosystems). Glyceraldehyde 3 phosphate dehydrogenase (GAPDH) was used as a loading control. The primers are listed in Additional file 1: Table S1.

### Western blot analysis

Proteins were separated by SDS-PAGE, transferred to polyvinylidene difluoride membranes (Millipore), blocked and probed with antibodies against β-catenin (1:1000; Cell Signaling Technology), p65 (1:1000; Cell Signaling Technology), GAPDH (1:4000; Santa Cruz), and FPGS (1:1000; Abcam). After washing, blots were incubated with horseradish peroxidase-conjugated secondary antibodies and visualized by super enhanced chemiluminescene (ECL) detection reagent (Appygen).

### Plasmid construction

Regions of FPGS gene promoter were generated by PCR using genomic DNA extracted from CCRF-CEM cells, and cloned into pGL3-basic vector (Promega). Primers used for this study are listed in Additional file 1: Table S1. All constructs were confirmed by Sanger sequencing.

### Assessment of apoptosis

Cells were treated with Annexin V Apoptosis Detection Kit APC (eBioscience) and analyzed using flow cytometry after treatment with MTX.

### Luciferase assay

The luciferase reporter assays were performed in triplicate by the Dual-Luciferase Reporter Assay Kit (TransDetect). HEK-293 cells were transiently transfected with luciferase reporter plasmids (pGL3-FPGS WT, pGL3-FPGS MUT-1, pGL3-FPGS MUT-2, and pGL3-Basic). Transfection efficiency was measured by co-transfection with a *Renilla* luciferase expression plasmid pRL-SV40 (Promega). The data were presented as the ratio of firefly luciferase activity to *Renilla* luciferase activity and results were presented as the mean ± SD.

### Chromatin immunoprecipitation (ChIP) and Re-ChIP

ChIP and Re-ChIP assays were performed using the ChIP-IT^®^ Express Chromatin Immunoprecipitation Kits and Re-ChIP-IT^®^ kits (Active Motif), according to the manufacturer’s instructions. Chromatin samples were immunoprecipitated with either anti-RelA antibody (Cell Signaling Technology) or normal mouse IgG (Cell Signaling Technology) as a negative control. Precipitated DNA was amplified by PCR using primers provided in the Additional file 1: Table S1. Non-immunoprecipitated chromatin fragments were used as an input control.

### Statistical analysis

The differences in results between groups were compared using Student’s t test. Data were expressed as mean ± SD. All statistical analyses were performed with the SPSS 16.0 Statistical program for Windows. *P* values less than 0.05 were considered significant.

## Results

### Reduction of β-catenin expression decreases MTX resistance in T-ALL cells

**Fig. 1:**
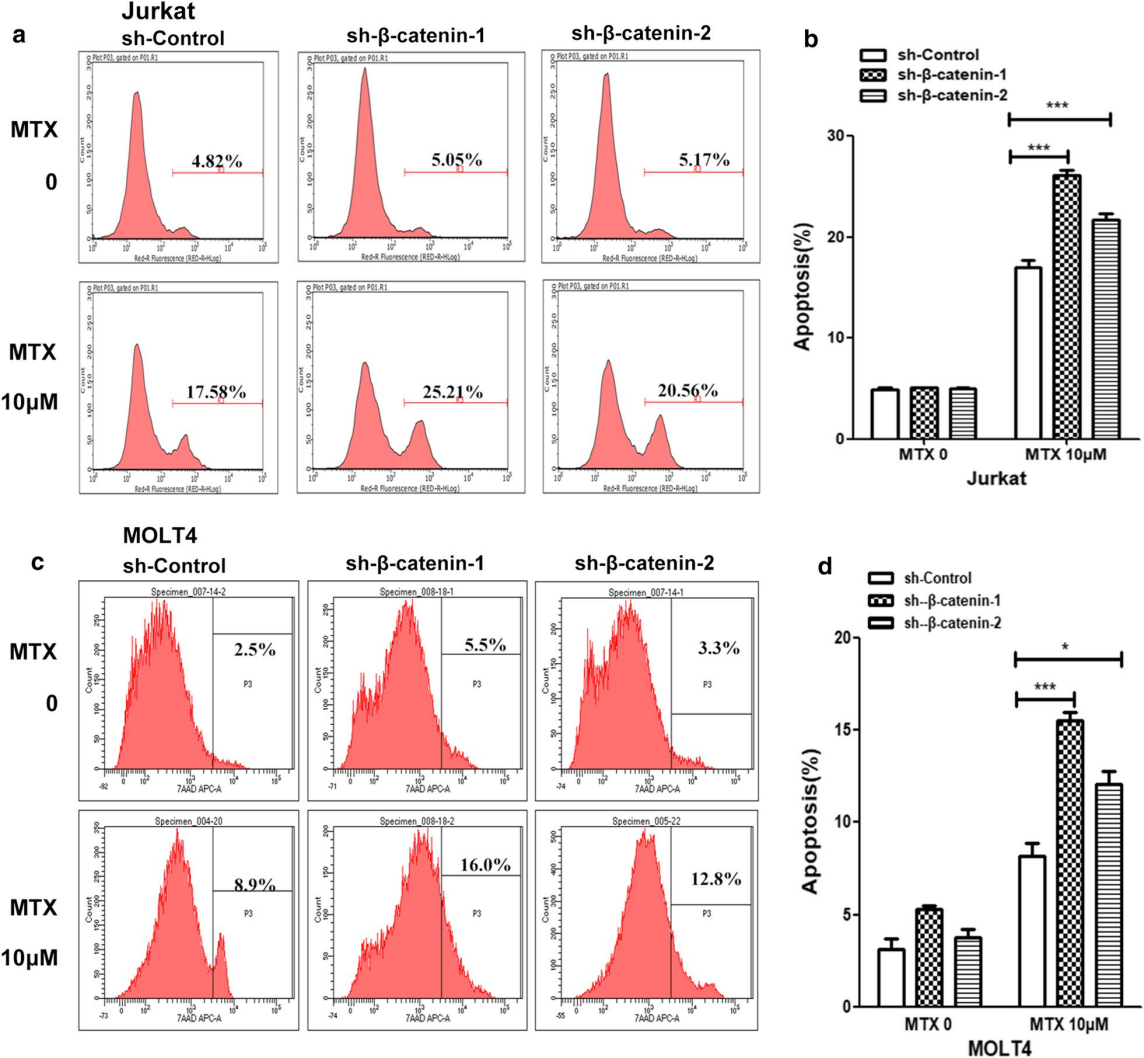
β-catenin inhibition promoted MTX resistance. **a** Flow cytometry data analysis of apoptotic cells by Annexin V-APC staining in Jurkat cells. After transfection of RNAi lentivirus, Jurkat cells were treated with 10 μM MTX for 24 h. **b** The percentage of apoptotic cells significantly increased in the two sh-β-catenin groups, compared with the control group in Jurkat cells. **c** Flow cytometry data analysis of apoptotic cells by Annexin V-APC staining in MOLT4 cells. After transfection of RNAi lentivirus, MOLT4 cells were treated with 10 μM MTX for 24 h. **d** The percentage of apoptotic cells significantly increased in the two sh-β-catenin groups, compared with the control group in MOLT4 cells. Columns, mean of 3 individual experiments; Bars, SD;*, *p *< 0.05, **, *p *< 0.01, ***, *p *< 0.001

To investigate the effect of β-catenin on MTX resistance, Jurkat and MOLT4 cells expressing high levels of β-catenin (Additional file 2: Figure S1) were transfected with β-catenin RNAi lentivirus for 48 h and then treated with 10 μM MTX for an additional 24 h. Flow cytometric results showed that the percentage of apoptosis cells were much higher in the β-catenin RNAi groups, compared with the sh-Control group (Fig. 1). These data revealed that β-catenin expression promotes MTX resistance.

### β-catenin inhibits the protein level of FPGS in T-ALL cells

**Fig. 2:**
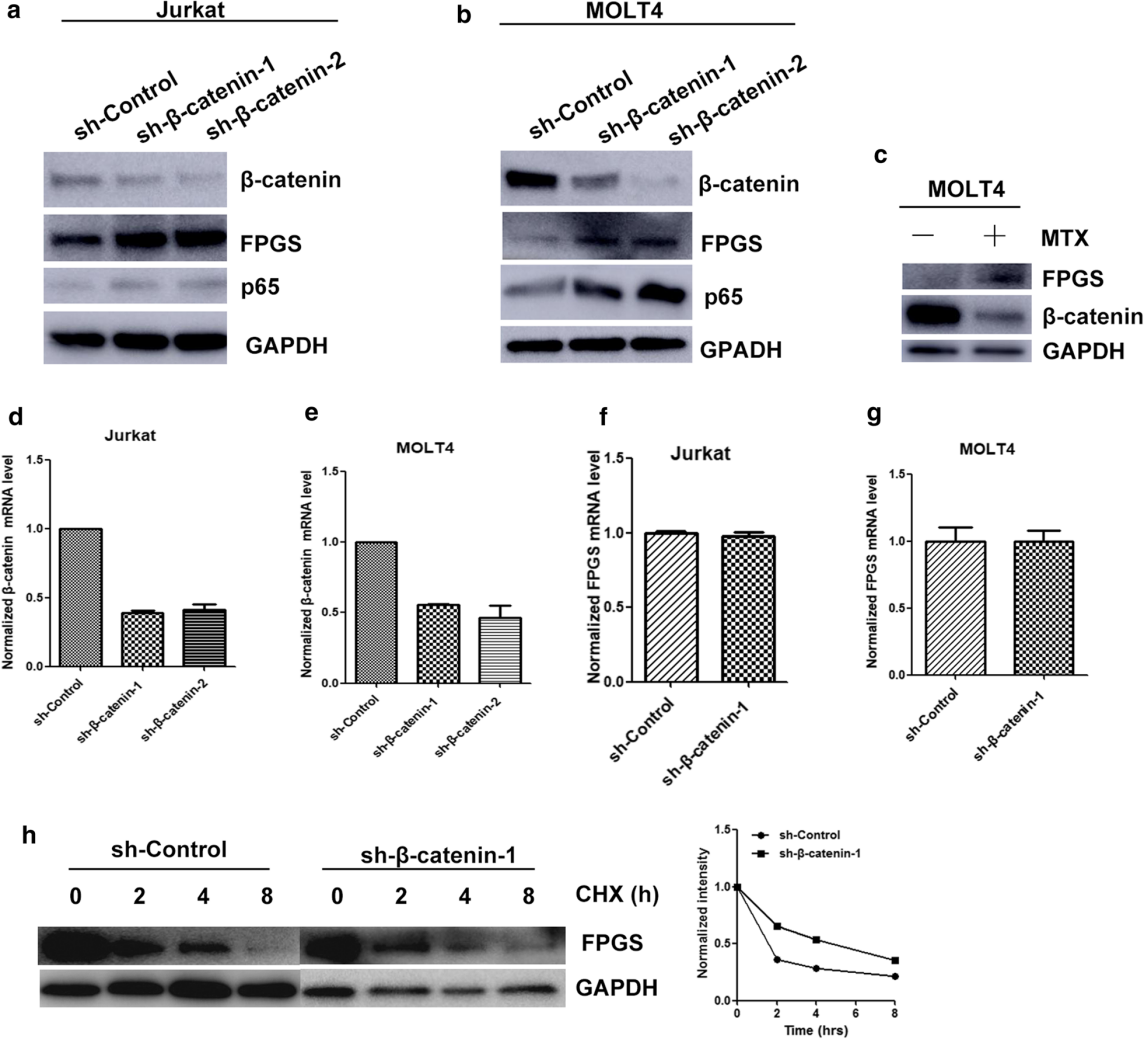
β-catenin regulated the protein expression of FPGS and NF-κB p65. **a** Western blot analysis of FPGS and p65 expression after β-catenin knockdown in Jurkat cells. **b** Western blot analysis of FPGS and p65 expression after β-catenin knockdown in MOLT4 cells. **c** Western blot analysis of FPGS and β-catenin expression after treatment with 10^−8^ M MTX for 2 weeks in MOLT4 cells. **d** The mRNA level of β-catenin in sh-Control or sh-β-catenin by qPCR in Jurkat cells. **e** The mRNA level of β-catenin in sh-Control or sh-β-catenin by qPCR in MOLT4 cells. **f** The mRNA level of FPGS in sh-Control or sh-β-catenin by qPCR in Jurkat cells. **g** The mRNA level of FPGS in sh-Control or sh-β-catenin by qPCR in MOLT4 cells. **h** Jurkat cells transfected with control shRNA or β-catenin-targeting shRNA were treated with CHX. The immunoblotting of FPGS and β-catenin (left) and the relative levels of FPGS (right) were quantifified

Given that FPGS is responsible for MTX activation and FPGS downregulation leads to resistance to MTX [5–7], we tested whether inhibition of β-catenin would affect the expression of FPGS. Western blot results showed that the protein level of FPGS was significantly increased after β-catenin depletion (Fig. 2a, b), demonstrating that β-catenin may suppress FPGS protein expression in ALL cells. After treatment of MTX at the concentration of 10^−8^ M for 2 weeks, the protein expression level of FPGS increased and that of β-catenin decreased, which confirmed the regulation between them (Fig. 2c).

### β-catenin regulates FPGS expression through interacting with NF-κB p65

**Fig. 3:**
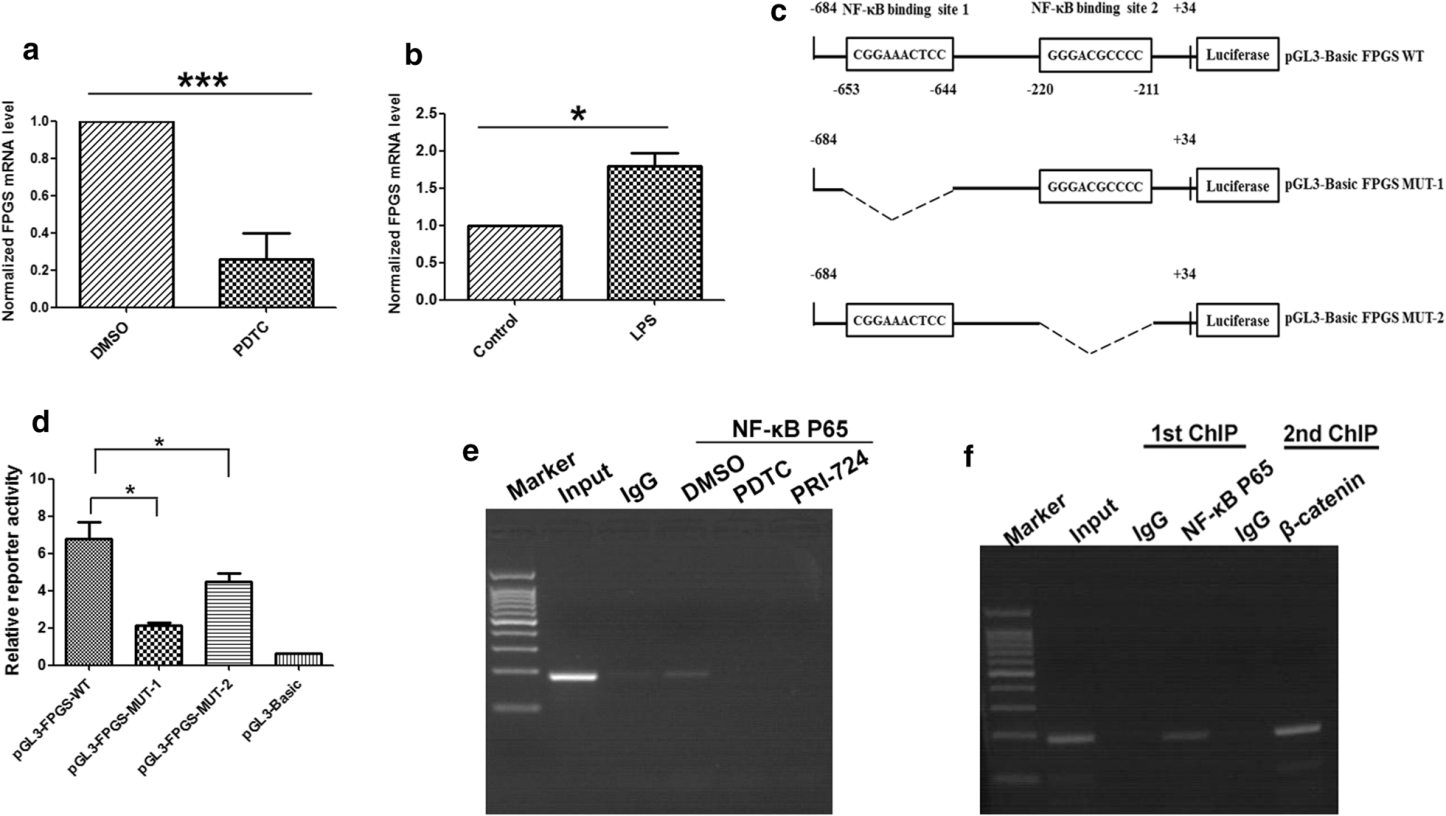
NF-κB p65 regulated FPGS transcription. **a** FPGS mRNA expression in Jurkat cells treated with DMSO or NF-κB inhibitor PDTC by qPCR. Columns, mean of 3 individual experiments; Bars, SD;*, *p *< 0.05, **, *p *< 0.01, ***, *p *< 0.001. **b** FPGS mRNA expression in Jurkat cells treated with H_2_O or NF-κB stimulant LPS by qPCR. Columns, mean of 3 individual experiments; Bars, SD;*, *p *< 0.05, **, *p *< 0.01, ***, *p *< 0.001. **c** Schematic diagram of NF-κB p65 binding sites in about 700 bp of the human *FPGS* promoter and the construction of MUT-1 and MUT-2. **d** Luciferase reporter assay in HEK-293 cells transfected with pGL3-Basic, pGL3-FPGS-WT, pGL3-FPGS-MUT-1, or pGL3-FPGS-MUT-1 plasmid. **e**, **f** ChIP and Re-ChIP assays in Jurkat cells

Previous study suggested that β-catenin can complex with NF-κB, inhibit NF-κB activity and repress its target genes in human colon and breast cancer cells [12]. Therefore, we hypothesized that β-catenin might regulate FPGS expression via NF-κB in ALL cells. As shown in Fig. 3a, after treatment of Jurkat cells with PDTC, a selective NF-κB inhibitor, the FPGS mRNA levels were significantly decreased. Moreover treatment with NF-κB stimulant LPS can dramatically increase the FPGS mRNA level (Fig. 3b). These data indicated that NF-κB p65 might regulate FPGS expression at the transcription level.

We next search the promoter region of FPGS and found two potential NF-κB p65 binding sites (Fig. 3c). To further confirm the transcriptional regulation of FPGS by p65, we subsequently generated a luciferase reporter vector, pGL3-FPGS WT, with *FPGS* promoter fragment (− 684 ~ 34). Deletion of the two p65 binding sites (MUT-1 for site 1 and MUT-2 for site 2), especially site 1 (− 653/644), significantly attenuated the *FPGS* promoter activity in HEK-293 (Fig. 3d). Moreover, ChIP assays showed that p65 directly bound to the − 653/644 region of the *FPGS* promoter, and the binding can be inhibited by NF-κB inhibitor PDTC and β-catenin inhibitor PRI-724 (Fig. 3e). These results clearly confirmed that FPGS is a direct transcriptional target of p65 in leukemia cells.

**Fig. 4:**
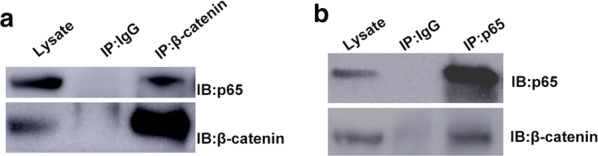
β-catenin interacted with NF-κB p65 in Jurkat cells. **a** Immunoprecipitation (IP) was performed with antibodies against β-catenin (normal IgG as a negative control). The complexes were then subjected to immunoblot as indicated. **b** IP was performed with antibodies against NF-κB p65 (normal IgG as a negative control). The complexes were then subjected to immunoblot as indicated

Consistent with previous reports, we found that β-catenin can also physically complex with NF-κB p65 (Fig. 4) in Jurkat cells. To futher explore the relationship between β-catenin and p65, we subsequently test the mRNA and protein levels of p65 after β-catenin knockdown. The mRNA level of p65 was not changed after β-catenin repression (data not shown). The protein level of p65 was increased (Fig. 2a, b). In addition, the protein levels of β-catenin and p65 were inversely correlated (Additional file 2: Figure S1) in ALL cell lines. To validate the interaction between p65 and β-catenin in the regulation of FPGS, we performed Re-ChIP assay (Fig. 3f). Results showed that p65 and β-catenin can bind to form a complex on the FPGS promoter in Jurkat cells.

Based on abovementioned data, β-catenin can interact with NF-κB p65, inhibit the protein levels of NF-κB p65 and its novel target gene FPGS in leukemia cells.

### β-catenin might also regulate FPGS protein stability

Since β-catenin depletion altered the protein level of FPGS without obvious change in the mRNA levels of FPGS (Fig. 2f, g), we further addressed whether the protein turnover of FPGS has been changed after β-catenin silencing. Results showed that knockdown of β-catenin significantly increased the stability of FPGS protein in cells treated with cycloheximide (CHX) (Fig. 2h). These results suggested that β-catenin might also be involved in the post-translational regulation of FPGS in T-ALL cells.

## Discussion

β-catenin is reported to be involved in the chemoreistance in many types of cancers [14, 17, 18], but there are few studies about its effects on drug resistance in ALL [19]. In this study, we have shown a novel role of β-catenin that it promotes MTX resistance in T-ALL cells. In molecular level, we found that β-catenin transcriptionally regulates FPGS expression by interacting with NF-κB. It can also regulate FPGS protein stability. Therefore, this study identified a novel β-catenin-NF-κB-FPGS pathway in MTX resistance in leukemia cells.

Previous studies showed that loss of FPGS activity is a mechanism of MTX resistance [5–7]. Transactivation and upregulation of FPGS have been reported to promote the cytotoxicity of MTX. Leclerc et al. [10] found that histone deacetylase 1 (HDAC1) is recruited by NFY-B and Sp1 proteins to the FPGS promoter and epigenetically regulate FPGS mRNA expression. Treatment with HDACi resulted in increased FPGS mRNA expression and higher intracellular accumulation of MTXPGs. We postulated that β-catenin may contribute to MTX resistance by regulating FPGS. Our data showed that after β-catenin inhibition the percentage of apoptotic cells significantly increased and the protein level of FPGS was increased, indicating that β-catenin might promote MTX resistance through inhibiting FPGS.

NF-κB is a transcription factor that controls multiple cellular processes in cancer including. It has been found to constitutively active in many solid tumors and hematological malignancies including ALL [20–22]. β-catenin has been shown to physically interact with NF-κB, inhibits its activity and represses the expression of NF-κB target gene, such as Fas, Traf1, and hiNOS [12, 23]. To test whether NF-κB is involved in the regulation between β-catenin and FPGS in leukemia cells, we performed coimmunoprecipitation assay and our result is in agreement with other findings that there is a interaction between β-catenin and p65. Moreover, our luciferase, ChIP, and Re-ChIP results indicated that FPGS is a novel target gene of NF-κB p65. These data demonstrated that NF-κB might be a mediator for the regulation of FPGS by β-catenin.

To further examine the relationship between β-catenin and NF-κB p65, we checked the mRNA and protein levels of p65 after β-catenin inhibition. There is no change on the mRNA level of p65, showing that β-catenin did not regulate the transcription of p65. Interestingly, we found that the protein level of p65 slightly increased after depletion of β-catenin and the protein expressions of the two molecules were inversely correlated in ALL cell lines (Additional file 2: Figure S1), which implicates that β-catenin might regulate NF-κB in the protein level. Previous studies have shown the cross regulation between β-catenin and NF-κB [20, 24–26]. β-catenin was found to form a complex with p65 and p50, leading to a decrease in NF-κB DNA binding and transactivation activity, and target gene expression. Studies revealed a negative effect of β-catenin on NF-κB activity and expression of downstream target genes in liver, breast, and colon cancer cells [12, 23, 27]. Such correlation was not seen in head and neck cancer [28]. Our results (Additional file 2: Figure S1 and Additional file 3: Figure S2) and previous study showed that β-catenin expressed higher in T-ALL samples and cell lines compared to B-ALL [24]. Moreover, it is reported that constitutive NF-κB activation is common in childhood ALL without any difference between T- and B-ALLs [22], implicating that β-catenin might not inhibit NF-κB activity in ALL, which is inconsistent with previous reports in colon, breast, and liver cancer [12, 23, 27]. It is possible that β-catenin regulates NF-κB in a different way in leukemia, and the regulation mechanisms need further investigation. There is a problem that the mRNA level of FPGS is not significantly changed after β-catenin knockdown, which seems contradictory. Although β-catenin interacts with p65 and regulates the transcription of FPGS, but the transcriptional regulation of FPGS is complex which involves many pathways [10, 11, 29–31], which might be a reason for this result. The CHX result showed that β-catenin might be also involved in the post-translational regulation of FPGS

Emerging evidence suggest that NF-κB can act as both tumor-promoting transcription factor and tumor suppressor [31–38]. In our study, p65 positively regulate FPGS transcription, and after inhibition of NF-κB activity by PDTC in Jurkat cells, the mRNA level of anti-apoptotic gene Bcl2 increased (Additional file 4: Figure S3), implicating a pro-apoptotic role of NF-κB in the MTX induced apoptosis in leukemia cells.

## Conclusions

In summary, our study reveals a novel signal pathway, β-catenin-NF-κB p65-FPGS, promoting MTX resistance in leukemia cells. The drug targeting β-catenin might be used in combination with MTX to overcome MTX resistance in T- ALL patients.

## Supplementary information

**Additional file 1: Table S1.** Primer sequences.

**Additional file 2: Figure S1.** β-catenin and NF-κB p65 expression in ALL cell lines. Western blot analysis of β-catenin and NF-κB protein expression in 5 ALL cell lines.

**Additional file 3: Figure S2.** β-catenin protein levels in childhood primary ALL samples. Western blot analysis of β-catenin protein expression in childhood primary leukemia cell.

**Additional file 4: Figure S3.** NF-κB inhibited the mRNA expression of Bcl2. Bcl2 mRNA expression in cells treated with DMSO or NF-κB inhibitor PDTC by qPCR

## Data Availability

The datasets used and/or analyzed during this study are available from the corresponding authors on reasonable request.

## Abbreviations

ALL: Acute lymphoblastic leukemia
MTX: Methotrexate
FPGS: Folypoly-γ-glutamate synthetase
MTXPGs: MTX polyglutamates
AML: Acute myeloid leukemia
FBS: Fetal bovine serum
PDTC: Pyrrolidine dithiocarbamate
BM: Bone marrow
ChIP: Chromatin immunoprecipitation
HDAC1: Histone deacetylase 1
