# Supplementary material for: β-catenin promotes MTX resistance of leukemia cells by down-regulating FPGS expression via NF-κB
Source: Cancer Cell Int. 2020 Jun 24;20:271. doi: 10.1186/s12935-020-01364-y (PMC7313175; doi:10.1186/s12935-020-01364-y)
Supplement: Supplementary file 1 — Additional file 1: Table S1. Primer sequences. [file 12935_2020_1364_MOESM1_ESM.docx]

**Table S1. Primer sequences**

| **gene** | **assay** | **sequences** |
| --- | --- | --- |
| FPGS | real-time PCR | F: CGAGGTTCGAGTCTTGCTC  R: TTCTGTTGGTCTGCGTTGC |
| GAPDH | real-time PCR | F: TGACTTCAACAGCGACACCCA  R: CACCCTGTTGCTGTAGCCAAA |
| FPGS-WT | Luciferase assay | F:CGGGGTACCCCAATGCTGGGAAGAGGGAG  R: CCGCTCGAGCTGCCAGGAATAGAGCGGC |
| FPGS-MUT-1 | Luciferase assay | F:ATCAATCATTTGAAGGGCTGCCT  R: TACCTCCTTCCCCTCTCCCT |
| FPGS-MUT-2 | Luciferase assay | F:GGAGCGTACACTCATAAACCT  R:CTCCCTTAGCGGGCACGCACT |
| FPGS | ChIP | F: GAGCAGAAGTCCAGCCAATG  R: TGCAAACAACCCACTACGG |
