# Supplementary figures and images for: β-catenin promotes MTX resistance of leukemia cells by down-regulating FPGS expression via NF-κB
Source: Cancer Cell Int. 2020 Jun 24;20:271. doi: 10.1186/s12935-020-01364-y (PMC7313175; doi:10.1186/s12935-020-01364-y)

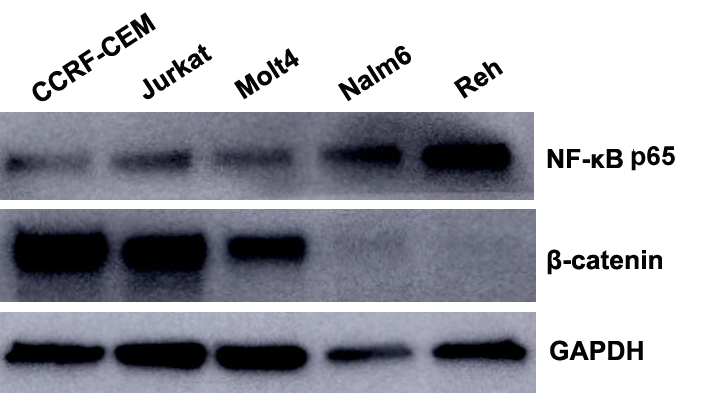

Supplement: Supplementary file 2 — Additional file 2: Figure S1. β-catenin and NF-κB p65 expression in ALL cell lines. Western blot analysis of β-catenin and NF-κB protein expression in 5 ALL cell lines. [file 12935_2020_1364_MOESM2_ESM.tif]

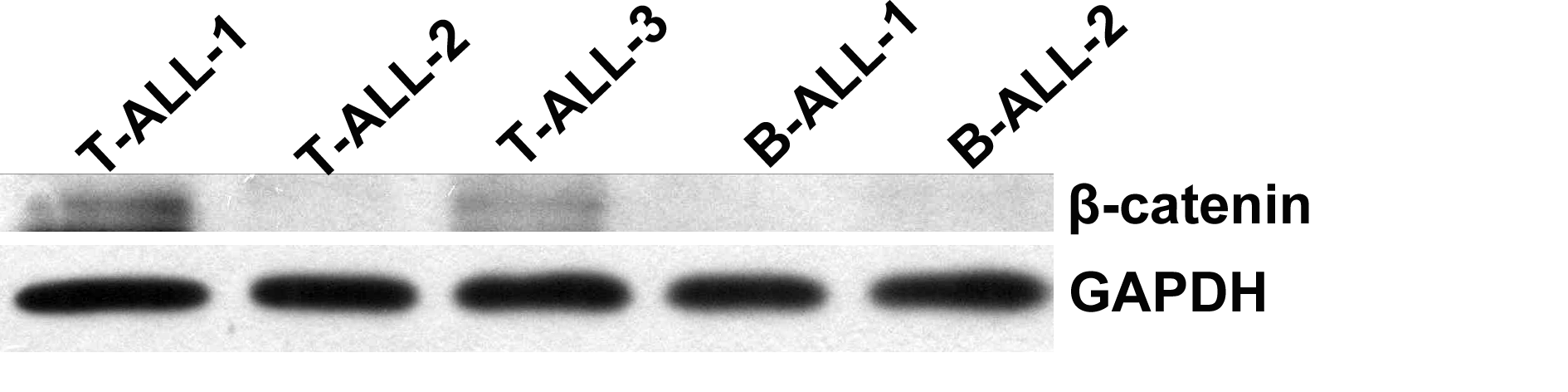

Supplement: Supplementary file 3 — Additional file 3: Figure S2. β-catenin protein levels in childhood primary ALL samples. Western blot analysis of β-catenin protein expression in childhood primary leukemia cell. [file 12935_2020_1364_MOESM3_ESM.tif]

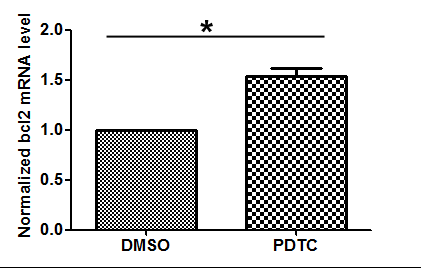

Supplement: Supplementary file 4 — Additional file 4: Figure S3. NF-κB inhibited the mRNA expression of Bcl2. Bcl2 mRNA expression in cells treated with DMSO or NF-κB inhibitor PDTC by qPCR [file 12935_2020_1364_MOESM4_ESM.tif]
